# Supplementary material for: Genetic regulation of OAS1 nonsense-mediated decay underlies association with risk of severe COVID-19
Source: medRxiv. 2021 Jul 13:2021.07.09.21260221. Preprint. [Version 1] doi: 10.1101/2021.07.09.21260221 (PMC8288155; doi:10.1101/2021.07.09.21260221)
Supplement: 1 [file NIHPP2021.07.09.21260221v1-supplement-1.pdf]

medRxiv preprint doi: https://doi.org/10.1101/2021.07.09.21260221; this version posted July 13, 2021. The copyright holder for this preprint (which was not certified by peer review) is the author/funder, who has granted medRxiv a license to display the preprint in perpetuity. This article is a US Government work. It is not subject to copyright under 17 USC 105 and is also made available for use under a CC0 license.

Supplementary Figures

Banday, Stanifer et al

Genetic regulation of *OAS1* nonsense-mediated decay underlies association with risk of severe COVID-19

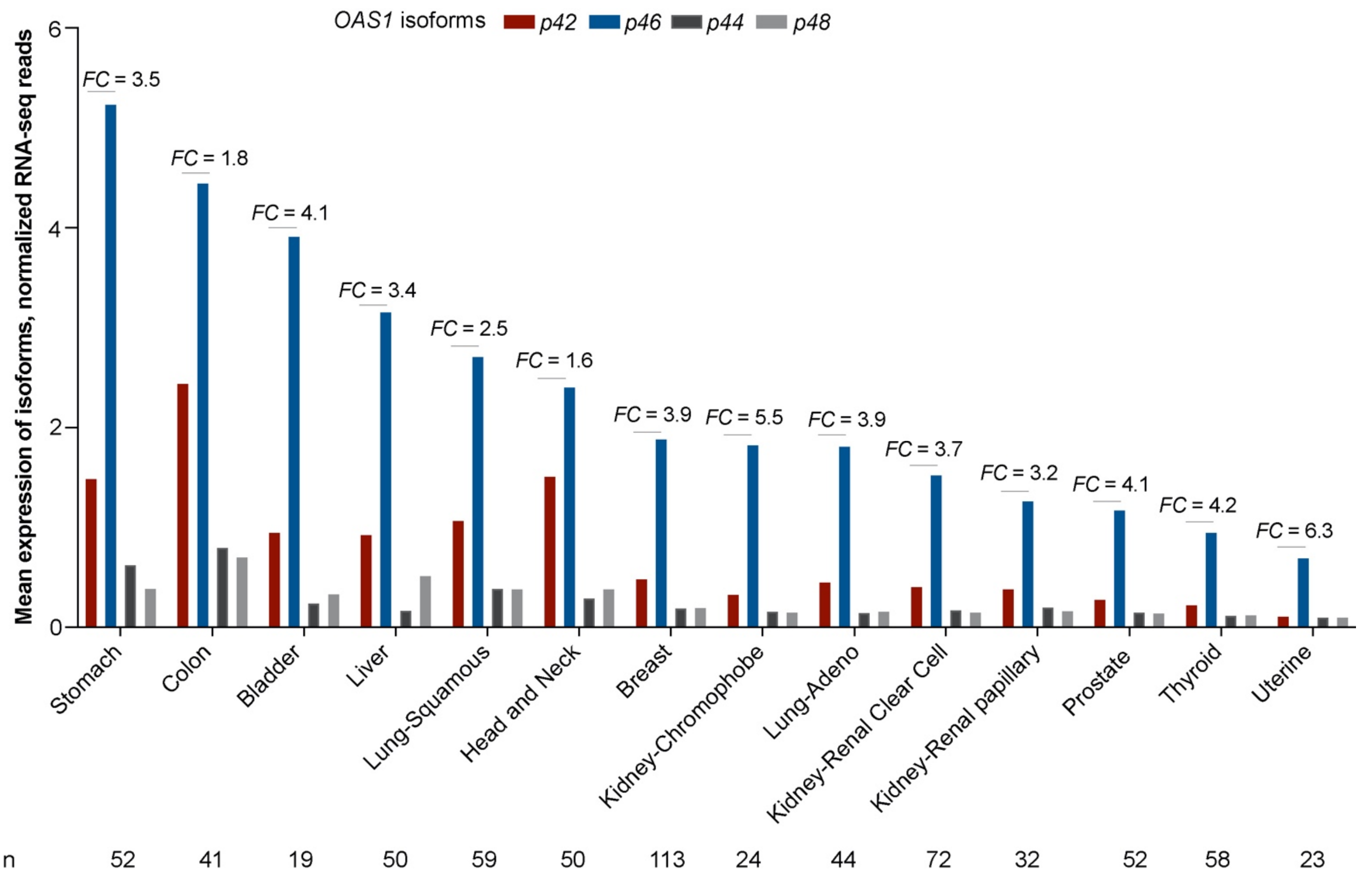

Figure S1. Mean expression levels of *OAS1* isoforms in adjacent normal tissues in TCGA. Expression of *OAS1* isoforms was analyzed in RNA-seq data in The Cancer Genome Atlas (TCGA), using tumor-adjacent normal tissues with RNA-seq data in ≥15 samples. RNA-seq reads for each *OAS1* isoform were calculated based on unique splice junctions (*OAS1-p44*, *p46,* and *p48*) or unique 3'UTR (*p42*). For normalization, the total number of RNA-seq reads for exon-exon junctions for *p44, p46* and *p48* was divided by 50 (length of an RNA-seq read) and unique RNA-seq reads for *p42* exon 5 by its length (317 bp). The mean expression levels of each isoform were calculated from all the samples with ≥3 RNA-seq reads. Overall, the mean expression of *OAS1-p46* is higher than *OAS1-p42*, with an average fold change (*FC*) of 3.9 across tissues.

medRxiv preprint doi: https://doi.org/10.1101/2021.07.09.21260221; this version posted July 13, 2021. The copyright holder for this preprint
(which was not certified by peer review) is the author/funder, who has granted medRxiv a license to display the preprint in perpetuity.
This article is a US Government work. It is not subject to copyright under 17 USC 105 and is also made available
for use under a CC0 license.

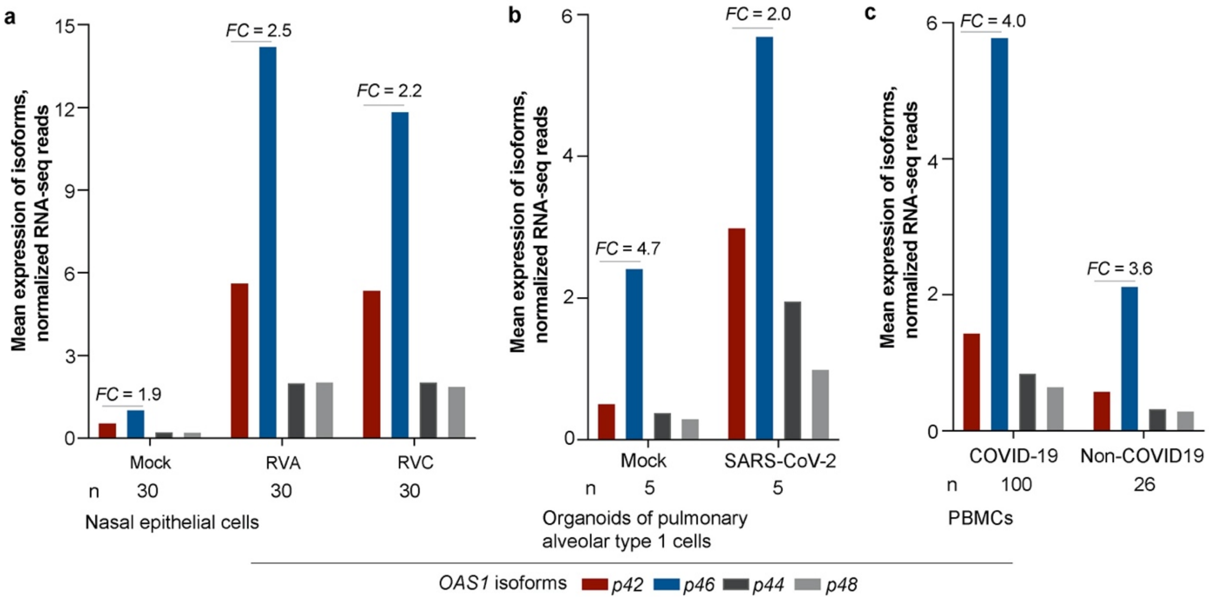

Figure S2. Mean expression levels of *OAS1* isoforms in nasal epithelial cells, pulmonary alveolar cells and PBMCs.

Expression of *OAS1* isoforms was analyzed in RNA-seq data of **a**) nasal epithelial cells (SRA PRJNA627860), **b**) organoids of pulmonary alveolar type 1 cells (SRA PRJNA673197), and **c**) PBMCs (SRA PRJNA660067). The bar graphs show the mean expression levels of each *OAS1* isoform normalized as described in **Figure S1**. The expression of *OAS1-p46* is higher than *OAS1-p42* in all cell types and conditions tested.

medRxiv preprint doi: https://doi.org/10.1101/2021.07.09.21260221; this version posted July 13, 2021. The copyright holder for this preprint (which was not certified by peer review) is the author/funder, who has granted medRxiv a license to display the preprint in perpetuity. This article is a US Government work. It is not subject to copyright under 17 USC 105 and is also made available for use under a CC0 license.

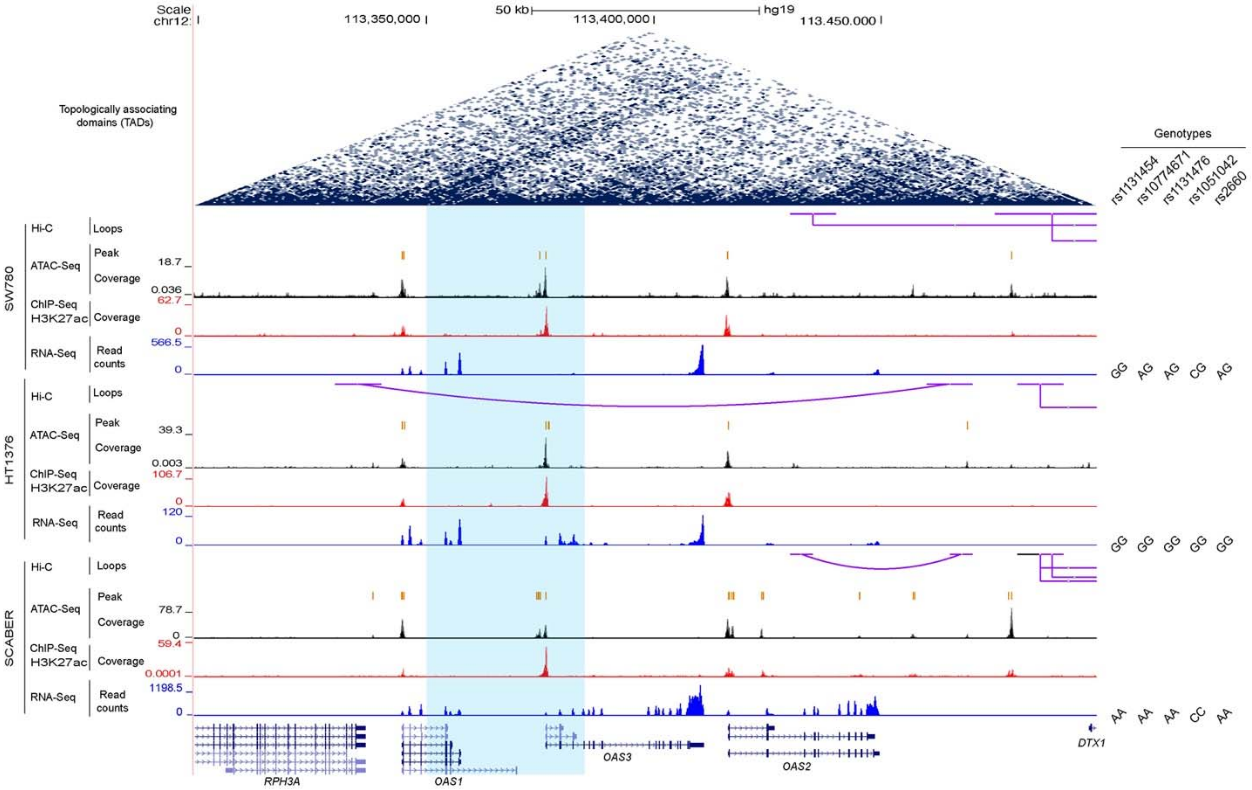

Figure S3. Multi-omics profile of the *OAS1/OAS3/OAS2* genomic region.

Multi-omics data for genome-wide Hi-C, ATAC-seq, H3K27ac ChIP-seq, and RNA-seq of three bladder cancer cell lines (SRA PRJNA623018) were analyzed and visualized using UCSC genome browser. *OAS1*, *OAS2*, and *OAS3* expression was observed in all samples, but there is no evidence of open chromatin, enhancer activity, or chromatin interactions within the LD block (blue shading) that includes top 125 variants associated with hospitalized vs. non-hospitalized COVID-19 in Europeans (**Figure 1**).

medRxiv preprint doi: https://doi.org/10.1101/2021.07.09.21260221; this version posted July 13, 2021. The copyright holder for this preprint
(which was not certified by peer review) is the author/funder, who has granted medRxiv a license to display the preprint in perpetuity.
This article is a US Government work. It is not subject to copyright under 17 USC 105 and is also made available
for use under a CC0 license.

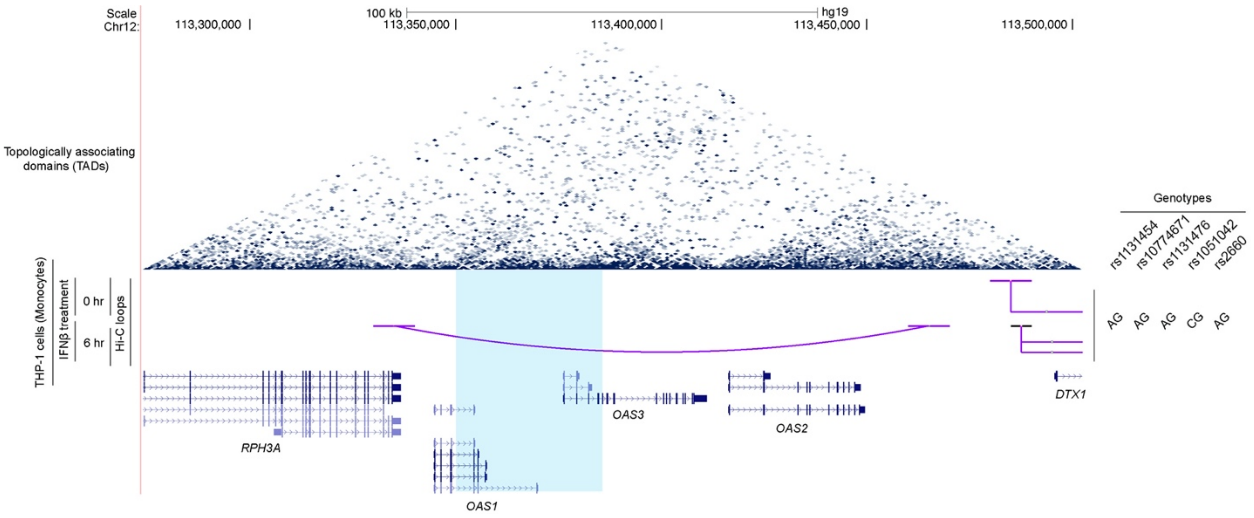

Figure S4. **Hi-C chromatin interaction analysis in the *OAS1/OAS3/OAS2* genomic region.**

Chromatin interaction (Hi-C) data for the THP-1 monocytic cell line untreated and treated with IFNβ for 6 hrs (SRA PRJNA401748) were analyzed and visualized using UCSC genome browser. No chromatin interactions were detected with the LD block (blue shading) that includes top 125 variants associated with hospitalized vs. non-hospitalized COVID-19 in Europeans (**Figure 1**).

medRxiv preprint doi: https://doi.org/10.1101/2021.07.09.21260221; this version posted July 13, 2021. The copyright holder for this preprint (which was not certified by peer review) is the author/funder, who has granted medRxiv a license to display the preprint in perpetuity.
This article is a US Government work. It is not subject to copyright under 17 USC 105 and is also made available for use under a CC0 license.

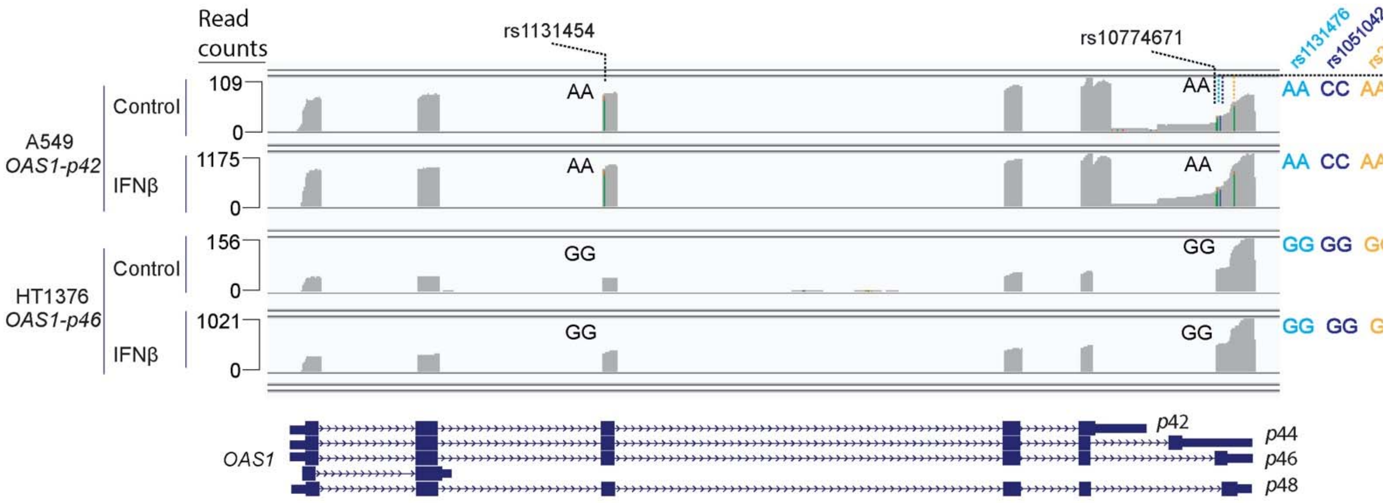

Figure S5. Long-read sequencing of *OAS1* transcripts in A549 and HT1376 cells with and without IFNβ treatment.

The A549 (rs10774671-AA, *OAS1-p42*) and HT1376 (rs10774671-GG, *OAS1-p46*) cells were treated with IFNβ or PBS (control) for 48 hrs. Total RNA was harvested and subjected to long-read sequencing with Oxford Nanopore. In both cell lines, the expression of *OAS1* isoforms was strongly induced by IFNβ treatment. The IGV plots show auto-scaled profiles with read counts indicating maximum coverage in the window. Genotypes of transcribed variants are indicated.

medRxiv preprint doi: https://doi.org/10.1101/2021.07.09.21260221; this version posted July 13, 2021. The copyright holder for this preprint (which was not certified by peer review) is the author/funder, who has granted medRxiv a license to display the preprint in perpetuity.
This article is a US Government work. It is not subject to copyright under 17 USC 105 and is also made available for use under a CC0 license.

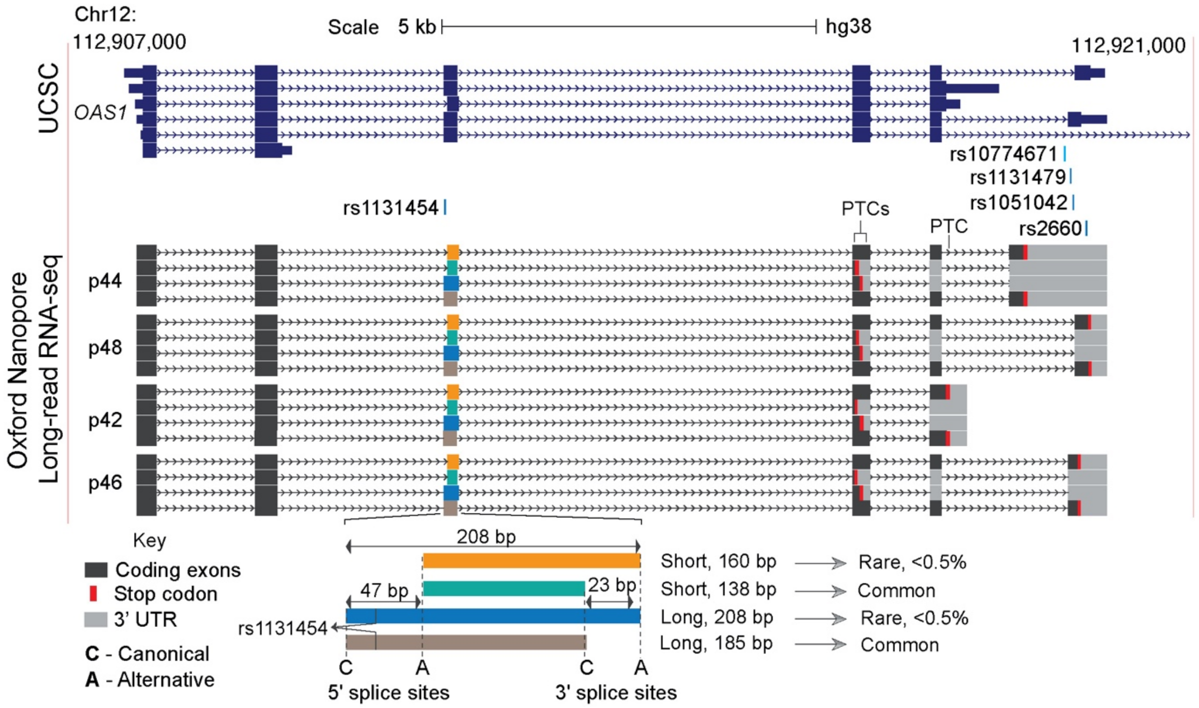

Figure S6. Long-read sequencing of *OAS1* transcripts demonstrates functional consequences of alternative splicing of exon 3.

UCSC genome browser view shows the known *OAS1* transcripts and full-length sequences of *OAS1* isoforms determined by long-read RNA-seq with Oxford Nanopore (**Figure S5**). Splicing patterns of *OAS1* exon 3 depend on two acceptor and two donor splice sites that define the Short and Long exon 3 isoforms. Two common exon 3 isoforms are Short (138 bp) and Long (185bp), and rare (<5% reads) exon 3 isoforms are Short (160 bp) and Long (208 bp). The inclusion of common Short (138 bp) or rare Long (208 bp) exon 3 isoforms results in premature termination codons (PTC) in exon 4. The stop codon of canonical *p42* isoforms presented by rare Short (160 bp) and common Long (185 bp) isoforms is located within exon 5 and functions as a PTC due to the inclusion of additional exons creating *p44* or *p48* isoforms. All transcripts with PTC are targeted by nonsense-mediated decay (NMD) with a variable extent of degradation depending on specific isoforms.

medRxiv preprint doi: https://doi.org/10.1101/2021.07.09.21260221; this version posted July 13, 2021. The copyright holder for this preprint (which was not certified by peer review) is the author/funder, who has granted medRxiv a license to display the preprint in perpetuity. This article is a US Government work. It is not subject to copyright under 17 USC 105 and is also made available for use under a CC0 license.

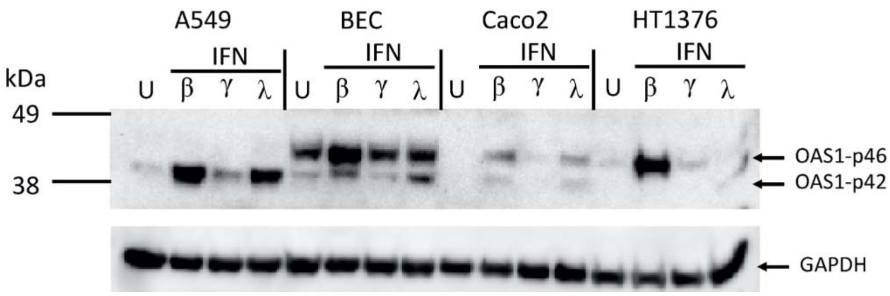

Figure S7. Western blot analysis of expression of endogenous OAS1-p42 and p46 protein isoforms in human cell lines.

Four cell lines with different genotypes of rs10774671: A549 (AA), primary bronchial epithelial cells BEC (AG), Caco2 (AG), and HT1376 (GG) were treated with interferons – IFNβ (2ng/mL), IFNγ (2ng/mL) or IFN-λ3 (100 ng/mL) for 48 hrs. Similar amounts of protein lysates were used for Western blot with C-terminal OAS1 antibody. Only two isoforms, OAS1-p42 and p46, were detected, with p46 protein being dominant in heterozygous cell lines. In homozygous cell lines, the protein expression of p42 and p46 appeared comparable after IFN treatment.

medRxiv preprint doi: https://doi.org/10.1101/2021.07.09.21260221; this version posted July 13, 2021. The copyright holder for this preprint (which was not certified by peer review) is the author/funder, who has granted medRxiv a license to display the preprint in perpetuity. This article is a US Government work. It is not subject to copyright under 17 USC 105 and is also made available for use under a CC0 license.

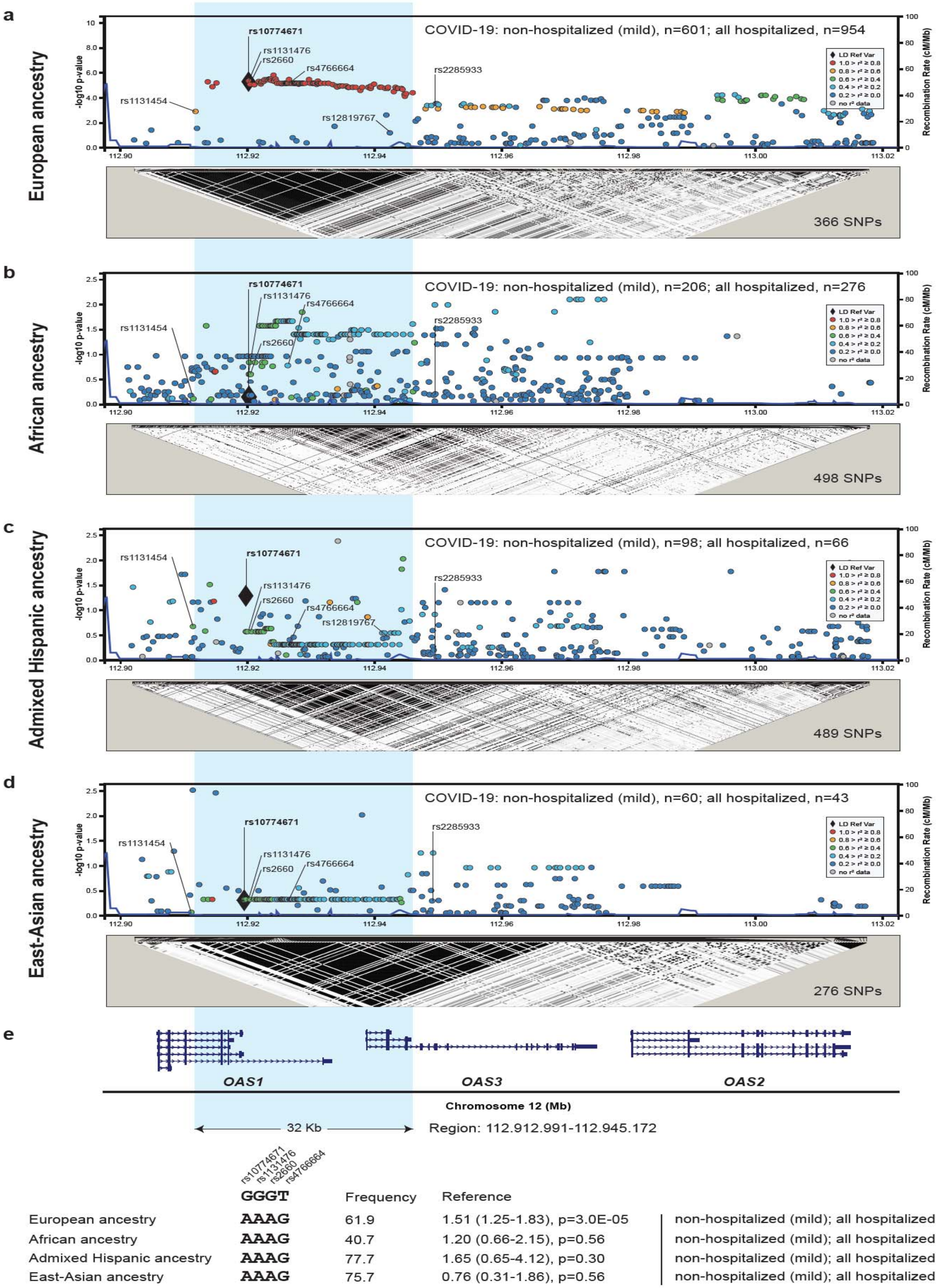

	rs10774671 rs1131476 rs2660 rs4766664<br>GGGT	Frequency	Reference	
European ancestry	AAAG	61.9	1.51 (1.25-1.83), p=3.0E-05	non-hospitalized (mild); all hospitalized
African ancestry	AAAG	40.7	1.20 (0.66-2.15), p=0.56	non-hospitalized (mild); all hospitalized
Admixed Hispanic ancestry	AAAG	77.7	1.65 (0.65-4.12), p=0.30	non-hospitalized (mild); all hospitalized
East-Asian ancestry	AAAG	75.7	0.76 (0.31-1.86), p=0.56	non-hospitalized (mild); all hospitalized

medRxiv preprint doi: https://doi.org/10.1101/2021.07.09.21260221; this version posted July 13, 2021. The copyright holder for this preprint
(which was not certified by peer review) is the author/funder, who has granted medRxiv a license to display the preprint in perpetuity.
This article is a US Government work. It is not subject to copyright under 17 USC 105 and is also made available
for use under a CC0 license.

Figure S8. Association analyses within the chr12q24.13 region in relation to COVID-19 outcomes in patients of diverse ancestries.
Association analyses and LD plots (r2) in patients with non-hospitalized (mild) vs. hospitalized COVID-19 in patients of **a**) European ancestry (n=1555); **b**) African ancestry (n=483); **c**) Admixed Hispanic ancestry (n=166); and **d**) East-Asian ancestry (n=103). **e**) location of *OAS1*, *OAS3,* and *OAS2* reference transcripts. Haplotype analysis of four core variants within the 32 Kb LD block (hg38: chr12:112912991-112945172), marked by blue shading, tagging the Neandertal haplotype in Europeans. Association results comparing the main risk vs. main non-risk (Neandertal) haplotypes. Full association results for individual variants and haplotypes are provided in **Tables S4-S8.**
